# Can We Predict Personality in Fish? Searching for Consistency over Time and across Contexts

**DOI:** 10.1371/journal.pone.0062037

**Published:** 2013-04-16

**Authors:** Maria Filipa Castanheira, Marcelino Herrera, Benjamín Costas, Luís E. C. Conceição, Catarina I. M. Martins

**Affiliations:** 1 CCMAR-CIMAR L.A., Centro de Ciências do Mar, Universidade do Algarve, Faro, Portugal; 2 IFAPA Agua del Pino., Instituto de Investigación y Formación Agraria y Pesquera, Cartaya, Spain; 3 CIIMAR-CIMAR L.A., Centro Interdisciplinar de Investigação Marinha e Ambiental, Universidade do Porto, Porto, Portugal; VIB & Katholieke Universiteit Leuven, Belgium

## Abstract

The interest in animal personality, broadly defined as consistency of individual behavioural traits over time and across contexts, has increased dramatically over the last years. Individual differences in behaviour are no longer recognised as noise around a mean but rather as adaptive variation and thus, essentially, raw material for evolution. Animal personality has been considered evolutionary conserved and has been shown to be present in all vertebrates including fish. Despite the importance of evolutionary and comparative aspects in this field, few studies have actually documented consistency across situations in fish. In addition, most studies are done with individually housed fish which may pose additional challenges when interpreting data from social species. Here, we investigate, for the first time in fish, whether individual differences in behavioural responses to a variety of challenges are consistent over time and across contexts using both individual and grouped-based tests. Twenty-four juveniles of Gilthead seabream *Sparus aurata* were subjected to three individual-based tests: feed intake recovery in a novel environment, novel object and restraining and to two group-based tests: risk-taking and hypoxia. Each test was repeated twice to assess consistency of behavioural responses over time. Risk taking and escape behaviours during restraining were shown to be significantly consistent over time. In addition, consistency across contexts was also observed: individuals that took longer to recover feed intake after transfer into a novel environment exhibited higher escape attempts during a restraining test and escaped faster from hypoxia conditions. These results highlight the possibility to predict behaviour in groups from individual personality traits.

## Introduction

In animals, individuals differ consistently in several aspects of their behaviour [1]–[3]. These individual differences may reflect distinct coping styles, behavioural syndromes, personalities or temperament. All these concepts embrace a similar definition which is a suite of correlated traits that are consistent across time and context [4]. In fish, two major personality types are recognised: proactive (active coping or bold or ‘fight-flight’) and reactive (passive coping or shy or ‘non-aggressive’). Proactive individuals create routines and seem to have a high level of active avoidance, locomotor activity and low flexibility in behavioural responses when faced with challenges, this pattern being the opposite for reactive individuals [4]–[6]. In addition, proactive individuals exhibit typical physiological and neuroendocrine characteristics such as lower hypothalamus-pituitary-interrenal (HPI) activity [7] and lower HPI reactivity [8] as compared to reactive individuals. In this paper personality traits are defined as physiological and behavioural responses to environmental changes which manifest as correlated trait-clusters [9].

The importance of understanding individual variation in fish has been shown to have implications in a wide range of fields including behavioural ecology [1], [3], neurosciences [10] aquaculture [11]–[13], welfare [8], [14], health and diseases susceptibility [15], [16], performance traits [8], [17] and interpretations of molecular data [10], [18], [19].

Fish are increasingly used as comparative models to uncover many of the fundamental question underlying the origin and implications of coping styles. Consequently, there is a growing interest on studying fish personality. Thus, while the importance of comparative studies to animal coping styles research is recognised [20], there is a lack of basic information that underlines the existence of personality in a particular species. Such information includes to which extent observed individual differences are consistent over time and predictive of other behaviours measured in different contexts. Consistency is used to describe a behavioural measure that is predictable across time and/or contexts. Even if the intensity of the behaviour changes, the rank position in relation to others, remains the same [1], [21]. A recent study using selected lines of rainbow trout (*Oncorhynchus mykiss*), proactive and reactive individuals were shown to exhibit consistency over a period of 7 days in traits associated to coping styles, feeding responses, presence of a novel object, aggressiveness and confinement [22]. Most of the studies on coping styles characterization have been done on selected fish lines which raises the question whether similar consistency responses can be observed in non-selected populations.

Another drawback of fish personality studies is the fact that the majority of tests developed are based on individually-housed animals [7], [23]–[25]. Individuals may differ in the interpretation of housing condition and consequently present distinct motivational states [26]. In addition, sociability has been shown to be a personality dimension, also in fish, suggesting that the effect of isolation can differ between individuals with different personality. Grouped-based tests may therefore have an added value when characterizing personality traits in fish. However, personality traits may also vary with social context [27] and phenomena such as facilitation may influence the results [28]. To the best of our knowledge no study has ever addressed personality traits in fish using both individual and group based screening tests.

Here, we investigate whether individual differences in behavioural responses to a variety of challenges can be used to assess personality in fish. Several tests were developed and repeated twice: feed intake recovery in a novel environment, novel object, restraining, risk-taking and hypoxia. These tests focus on one personality dimension: the exploration-avoidance [20,29 also as a review of the other personality dimensions in fish]. Gilthead seabream (*Sparus aurata*) was used as our model specie, as it is widely used in research due to its robustness and well known biology and behaviour. It is also ranked second as the most important European farmed fish [30].

## Materials and Methods

All experiments described were conducted in accordance with the Guidelines of the European Union Council (86/609/EU) and Portuguese legislation for the use of laboratory animals, and under a "Group-1" licence from the Veterinary Medicines Directorate, the Portuguese competent authority for the protection of animals, Ministry of Agriculture, Rural Development and Fisheries, Portugal. Permit number 0420/000/000-n.99-09/11/2009. At the end of the experimental procedures, individuals used in this study were kept under group conditions (11.2 kg m^−3^) and optimal water and feeding conditions as they will be used in another study that aims at looking at consistency of personality over longer time periods.

### Experimental animals, housing and feeding

Twenty-four juveniles of Seabream, *Sparus aurata*, with an initial body weight of 49.31±7.25 g (means±SD) were used as experimental animals. All animals were obtained from a seabream producer (MARESA Mariscos de Esteros SA, Huelva, Spain) and were kept in stock groups until the start of the experiment. Individuals were individually PIT-tagged (Trovan®, Netherlands) one week before the start of the experimental procedures. Water temperature (19.8±2.1°C), salinity (33.8±2.4 ‰), dissolved oxygen (98.4±2.8%), NO_2_-N (0.0±0.0 mg L^−1^) and NO_3_-N (0.0±0.0 mg L^−1^) were checked daily. A 12L: 12D photoperiod was maintained with day break set at 8:00 h. Fish were fed with automatic feeders, with commercial diet (Aquagold 2 mm, Sorgal SA, Portugal; 44% crude protein, 14% crude fat, 8% ash, 2.5% crude fibres, 1.0% phosphorus). The same feed and photoperiod was used during the experimental procedures.

### Personality screening

**Figure 1:**
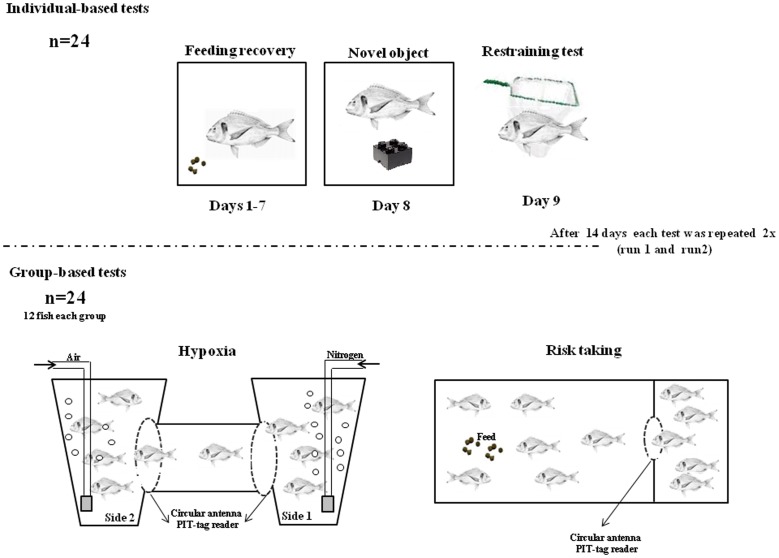
Schematic representation of the experimental set-up used to determine personality in Gilthead seabream *Sparus aurata*. Daily feed intake recovery on isolated fish (n = 24), was recorded during 7 days. On day 8 and 9the same fish were submitted to novel object test and net restraining test, respectively. Each test was repeated twice (run 1 and run 2) with an interval between runs of 14 days. Individually-based tests were run first (both run 1 and 2) followed by the grouped-based tests.

Each fish was subjected to the following tests: 1) Feeding recovery in a novel environment (adapted from Øverli et al. [23], 2) Novel object (adapted from Frost et al. [31], 3) Restraining (adapted from Arends et al. [32], Silva et al. [7] and Martins et al. [33] 4) Hypoxia (adapted from Laursen et al. [34] and 5) Risk-taking (adapted from Huntingford et al. [35]. Tests 1–3 were individually-based while tests 4 and 5 were grouped-based (see Figure 1). Each test was repeated twice (run 1 and run 2) with an interval between runs of 14 days. Individually-based tests were carried out first (both run 1 and 2) followed by the grouped-based tests. Between individual and groups-based tests, fish were kept in groups of 12 fish. These groups were maintained during the group testing.

### Individual-based tests

Fish were housed individually in a 40 L glass aquarium (37 cm length ×40 cm width ×40 cm depths) in an open water circuit during 9 days. The water flow rate was 60 L.h^−1^, nearly 1.5 renovations *per* hour. Water temperature (19.3±2.1°C), salinity (33.8±2.4 ‰), dissolved oxygen (98.1±1.3%), NO_2_-N (0.0±0.0 mg L^−1^) and NO_3_-N (0.0±0.0 mg L^−1^) were checked daily.

#### Feeding recovery test

The feeding recovery test consisted of following daily feed intake recovery in fish housed in isolation for 7 days. Fish (n = 24) were fed *ad libitum*, by hand, twice per day (09:30 and 15:30) using the same diet mentioned before. The order of feeding was randomized every meal. Five pellets were added at the start of feeding and the number of pellets eaten by each fish was noted and replaced by new ones as soon as they were consumed. Feeding continued for a maximum of 1 h, after which the remaining pellets were collected and counted. Feeding recovery was determined as following: feeding latency (time in seconds taken by each fish to consume the first pellet); total feeding time (total time in seconds taken by each fish to consume all pellets until apparent satiation); number of feeding acts (number of times an individual approached the pellets resulting in feed consumption), number of feeding days (number of days that result on feed intake) and feed intake (% BW^−1^).

#### Novel object test

The novel object test (day 8, after onset of isolation) consisted of a Lego® brick (3 cm length ×3 cm width ×2.3 cm height – used during the 1^st^ run) or a table tennis ball filled with sand (2 cm radius – used during the 2^nd^ run) that were dropped suddenly in the middle of the tank. The bottom of the test tanks were divided into three distinct zones: 5 and 10 cm radius around the novel object and the remaining area, which were marked with a text marker on the bottom of the tank. Fish behaviour was video recorded (SONY, DCR-SR190E, Japan) for posterior analyses. Cameras were placed above the tanks. The observation period lasted 15 minutes and started immediately after the novel object was dropped in the tank. During the 15 min observation period the following parameters were measured: latency to enter the 5 cm and 10 cm radius areas (time in seconds taken by each fish to enter in each area) and the number of times fish entered in each area. The entrance in the area was defined when the snout of the fish was inside the area.

#### Restraining test

The net restraining test (day 9, after onset of isolation, last day of individually-based tests) consisted of holding each fish individually in an emerged net for three minutes [7], [32], [33]. While in the net the following behaviours were measured: latency to escape (time in seconds taken by each fish to show an escape attempt; escape attempt was defined as a elevation of the body from the net; number of escape attempts and total time spent on escape attempts (total time in seconds taken by each fish escaping since the first to the last escape attempts).

Blood samples were collected 30 minutes after the start of net restraining, according to Arends et al. [32]. Therefore, fish were quickly taken out from each tank at the same time and anaesthetized with 2-phenoxyethanol (1000 ppm, Sigma-Aldrich). Blood was withdrawn within 3 min from caudal vein using heparinised syringes and centrifuged at 2000× *g* for 20 minutes at room temperature. After centrifugation plasma was frozen in liquid nitrogen and stored at −80°C for cortisol analysis. After blood sampling individuals were weighed and identified.

### Group-based tests

#### Hypoxia test

**Figure 2:**
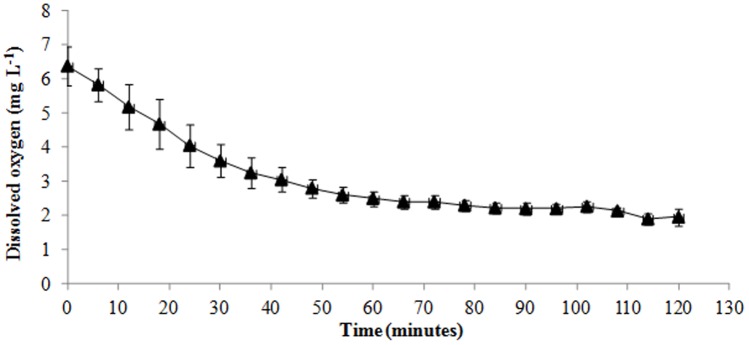
Decrease of dissolved oxygen in the water (DO) over a period of hypoxia test. Values are the mean of two runs for all the individuals.

The hypoxia test consisted of reducing the oxygen levels in one side of a two-chamber tank and measuring the escape behaviour from the hypoxia to the normoxia side. The tank was composed of two similar circular tanks (40 L) that were connected with a transparent plastic pipe (40 cm length ×6 cm radius). In the extremes of the connection pipe two circular antennas were placed, (diameter 100/125 ×20 mm Trovan®, Netherlands), to allow individual tracking of the fish passing through the pipe. Each side of the tank was equipped with water inflow, outflow and air stone supply. The connection pipe was closed with a removable door (13 cm length ×13 cm width) before the start of the test. Each group of fish (n = 12) were allowed to settle overnight in one side (side 1) before the start of the experiment. At the beginning of the experiment the water supply was stopped on both sides. Aeration on side 1 was turned off and replaced by nitrogen which leads to a gradual decrease in oxygen concentration (Figure 2). Afterwards, the door blocking the connection tube was removed and the circular antennas started to register the fish movement between sides. The dissolved oxygen in the water (DO) was measured by an Oxyguard handy probe (Handy Delta, USA). Figure 1 shows the DO decrease over time. During the hypoxia test, fish behaviour was video recorded (MicroVideo™ camera MCV2120-WP-LED, Canada) for posterior analyses. The following behaviours were measured: latency to escape hypoxia (time in seconds taken by each fish to escape hypoxia conditions); order of escape and number of returns (number of times an individual returns to the hypoxia side after being in the normoxia side). The hypoxia test was finalised when half of the fish escaped from the hypoxia side or when a concentration of 3 mg.L ^−1^ DO was reached.

#### Risk-taking test

The risk-taking test was done on a 300 L fibreglass tank (100 cm length ×60 cm width ×50 cm depth) separated in two distinct areas: safe and risk areas. The areas were divided using a solid plastic partition (2 mm thickness) with a hole (6 cm radius), connected to a circular antenna, diameter 100/125×20 mm (Trovan®, Netherlands) that allowed the identification of which fish passed through the hole and the time of each passage. The connection hole was closed with a removable door (13 cm length ×13 cm width). Each group of fish (n = 12) were allowed to settle overnight in the safe area before the start of the experiment. At the beginning of the experiment the door was removed and 10 pellets (6% BW^−1^) were released into the risk area every 5 minutes to stimulate fish going to the risk area. Fish behaviour was video recorded (MicroVideo™ camera MCV2120-WP-LED, Canada) for posterior measurement of: latency for risk-taking (time in seconds taken by each fish to enter the risk area); order of risk-taking and number of returns (number of times an individual returns to the safe area after being in the risk area). The risk-taking test was finalised when half of the fish entered in the risk area or 4.5 hour after the beginning of the experiment.

### Cortisol analyses

Plasma cortisol levels were measured with a commercially available competitive binding Coat-A-Count® Cortisol kit (SIEMENS Medical Solutions Diagnostics, USA) adapted from Irwin et al. [36]. Briefly, 50 ml of each sample to be assayed was transferred into an Ab-Coated tube and 1 ml of ^125^I Cortisol added. The tubes were then incubated for 45 min at 37°C in a water bath. The contents of all tubes were decanted, and allowed to drain for 5 min before being read on a gamma counter (2470 WIZARD^2^™, PerkinElmer TM, Inc., Belgium) for 1 min. A calibration curve was used to convert results from percent binding cortisol to concentration (ng ml^−1^). The Coat-A-Count cortisol antiserum cross-reacts: 100% with cortisol, 11.4% with 11-deoxycortisol, 0.98% with cortisone, 0.94% with corticosterone and 0.02% with progesterone.

### Data analysis

Statistical analyses were performed using SPSS 18.0 for windows. The results are expressed as mean±standard deviation (SD). Behaviours measured in each test were collapsed into first principal component scores using Principal Components Analysis (PCA). The correlation matrix was used to check multicollinearity, i.e., to identify variables that did not correlate with any other variable, or correlate very highly (*r* = 0.9) with one or more variables. Kaiser–Meyer–Olkin (KMO) test for sample adequacy was always greater than 0.5 and the Bartlett's test of sphericity was significant for all tests. The PC1 for run 1 and run 2 for each test was averaged and used to investigate cross-context relationships. Spearman correlation analyses were used after data failed to pass the normality Kolmogorov-Smirnov test. In addition, a two-step cluster analyses was performed using the PC1 average (from run 1 and 2) of the tests that revealed consistent responses over time (risk-taking and net restraining). An independent-samples T test, was used to verify differences between the generated clusters. Statistical significance was taken at p<0.05.

## Results

### Individual variation

Table 1 depicts the pronounced individual variation in different behavioural variables obtained for each test in Gilthead seabream *Sparus aurata* (n = 24).

**Table 1:** Mean±SD, minimum (Min.) and maximum (Max.) values of behavioural variables obtained for each test in Gilthead seabream *Sparus aurata* during all the experimental procedures (n = 24).

		Run 1			Run 2		
Behavioural context	Behaviours within each context	Mean±SD	Max.	Min.	Mean±SD	Max.	Min.
Feeding recovery	Lat feeding (sec)	2622.35±828.08	3600.00	898.71	2159.99±923.78	3567.21	852.43
	Total feeding time (sec)	480.49±559.93	1821.86	0	825.77±629.73	1996.14	0
	# feeding sessions	1.50±1.58	4.86	0	3.11±2.53	9	0
	Feed intake (% BW)	0.16±0.17	0.55	0	0.26±0.22	0.67	0
	# feeding days	3±2	6	0	3±2	7	1
Novel object	Lat 5 cm radius area (sec)	387.50±370.30	900.00	19.00	489.23±345.26	900.00	10.00
	#5 cm radius area	8±10	36	0	3±4	13	0
	Lat 10 cm radius area (sec)	207.08±282.57	900.00	19.00	298.27±285.03	900.00	2.00
	#10 cm radius area	13±11	43	0	8±8	26	0
Restraining	Lat escape (sec)	99.96±65.98	180.00	1.00	41.96±33.20	124.00	1.00
	# escapes	8±8	24	0	17±8	35	6
	Total escape time (sec)	8.71±10.25	38.00	0.00	15.65±10.05	43.00	2.00
Hypoxia	Hypoxia lat (sec)	7048.00±7378.00	16200.00	0.00	4167.00±4842.00	16200.00	1020.00
	# returns	4±7	24	0	7±8	23	0
	Hypoxia escape order	8±5	15	1	6±4	15	1
Risk taking	Risk latency (sec)	9323.00±6869.00	16200.00	300.00	7553.00±7897.00	16200.00	0.00
	# returns	1±2	11	0	4±8	29	0
	Risk escape order	8±5	15	1	8±5	15	1

### Consistency over time

The consistency over time in behavioural responses is shown in Table 2. There was a strong positive correlation between the behaviour in run 1 and 2 of the restraining (*r_s_* = 0.36, *p* = 0.01) and risk taking (*r_s_* = 0.53, *p*<0.001) tests. Feeding recovery (*r_s_* = 0.28, *p* = 0.06) and hypoxia (*r_s_* = 0.40, *p* = 0.06) showed a strong trend (*p* = 0.06) towards consistency over time while the novel object test (*r_s_* = −0.98, *p* = 0.66) did not result in consistent behavioural responses.

**Table 2:** Consistency over time (run 1 and run 2) of behavioural responses in Gilthead seabream *Sparus aurata* obtained during transfer into a novel environment, novel object, restraining, risk-taking and hypoxia tests (n = 24).

Consistency over time	Feeding Recovery Run2	Novel Object Run2	Restraining Run2	Risk taking Run2	Hypoxia Run2
**Feeding Recovery Run1**	*r_s_ = *0.28 *p = *0.06				
**Novel Object Run1**		*r_s_ = *−0.98 *p = *0.66			
**Restraining Run1**			*r_s_ = *0.36 *p = *0.01		
**Risk taking Run1**				*r_s_ = *0.53 *p = *0.00	
**Hypoxia Run1**					*r_s_ = *0.40 *p = *0.06

After the restraining test, the cortisol values were 36.17±32.54 ng ml^−1^ (means±SD) and varied between 6.2 ng ml^−1^ and 117.33 ng ml^−1^ in run 1 and were 40.87±27.52 ng ml^−1^ (means±SD) and varied between 9.9 ng ml^−1^ and 87.41 ng ml^−1^ in run 2. Cortisol responsiveness was not consistent over time (p>0.05). Behavioural responses during the restraining test were not correlated with cortisol responsiveness.

### Cross-context consistency: correlations between tests

**Figure 3:**
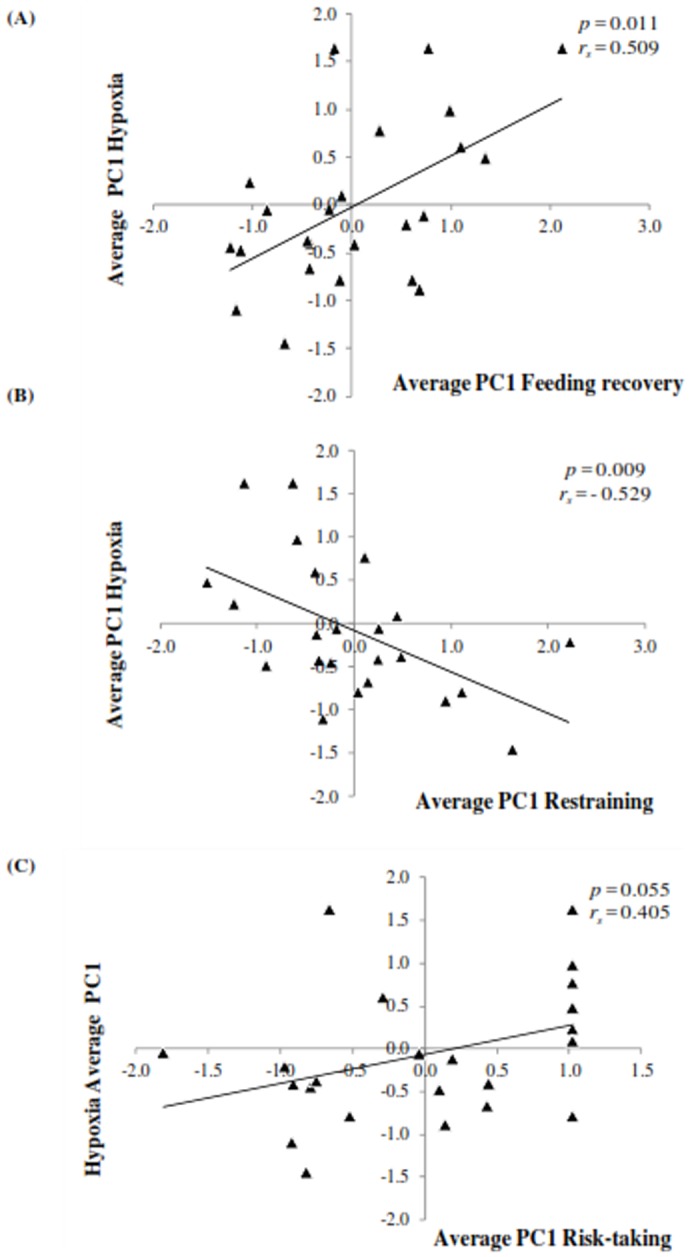
Relationship between the average PC1 behavioural score (from run1 and run2) during the hypoxia and feeding recovery (A – individuals with high hypoxia scores took longer to escape hypoxia conditions and resumed feed intake faster), net restraining (B - individuals with high hypoxia scores took longer to escape hypoxia conditions and escaped less during net restraining) and risk taking (C - individuals with high hypoxia scores took longer to escape hypoxia conditions and longer to take risks) tests on seabream *Sparus aurata* juveniles (n = 24).

The PCA loadings of each test used to generate a principal component score (PC1) to assess cross-context correlations are shown in Table 3. Figure 3 depicts the relationship between the average PC1 (run1 and run 2) for the behavioural responses observed during feeding recovery, restraining, hypoxia and risk taking test. Individuals that escaped faster from hypoxia, tried to escape more in a restraining test (*r_s_* = −0.53, *p* = 0.01), were more risk-takers (*r_s_* = 0.40, *p* = 0.05) and took longer to recover feed intake (*r_s_* = 0.51, *p* = 0.01) while in isolation.

**Table 3:** PCA loadings of within-context behavioural variables used to generate a principal component scores (PC1) in run 1 and run 2.

Behavioural context	Behaviours within each context	Loadings for PC1- RUN 1(component matrix)	% Variation explained	Loadings for PC1- RUN 2(component matrix)	% Variation explained
					
**Feeding recovery**	Latency feeding	−0.981	95.458	−0.959	88.058
	Total feeding time	0.978		0.948	
	Number feeding sessions	0.965		0.932	
	Feed intake	0.975		0.928	
	Number feeding days	0.986		0.926	
**Restraining**	Latency escape	−0.835	83.041	−0.773	59.431
	Number escapes	0.964		0.655	
	Total escape time	0.929		0.870	
**Hypoxia**	Hypoxia latency	0.963	76.208	0.904	74.598
	Number returns	−0.666		−0.751	
	Hypoxia escape order	0.957		0.925	
**Risk taking**	Risk latency	0.941	77.311	0.957	80.174
	Number returns	−0.729		−0.744	
	Risk escape order	0.950		0.967	

**Figure 4:**
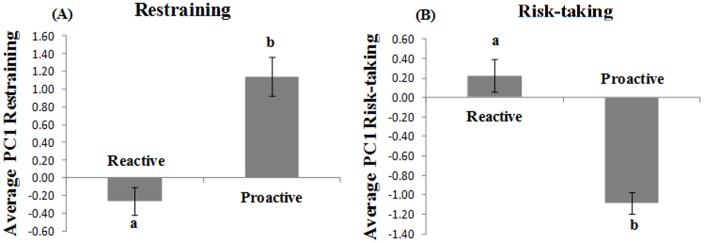
Distinct groups (Proactive (n = 20) and Reactive (n = 4)) generated after cluster analysis, based on restraining and risk-taking PC1 average. Different letters indicate significant differences (independent T-test): restraining (*p* = 0.05); risk-taking (*p* = 0.01)). A- Individuals with high restraining scores escaped more during net restraining. B- Individuals with high risks scores took longer to take risks.

Two groups were generated with the cluster analysis (proactive, n = 20 and; reactive, n = 4) based on restraining and risk-taking PC1 average. Figure 4 depicts the differences between proactive and reactive individuals showing that one of the clusters (which we call- Proactive individuals) escaped significantly more during restraining (*p* = 0.05) and were more risk-takers (*p* = 0.01) as opposed to the other cluster (Reactive individuals).

## Discussion

This study characterized for the first time fish personality considering both the consistency of behavioural differences over time and across contexts using a battery of individual and grouped-based tests. Among the different tests used, the escape behaviour during restraining and the risk taking behaviour showed the most consistent results. In addition, a relationship across contexts was found between hypoxia and feeding recovery, net restraining and risk taking tests.

Considering the consistency of behavioural responses over time, the escape response during a restraining test was shown to be the most repeatable: individuals showing lower latency to escape, higher number of escape attempts and spending more time escaping in run 1 showed a similar behaviour after 14 days when the test was repeated. Escaping behaviour during restraining or confinement has been used to discriminate coping styles in other animals, e.g pigs [37] and also in fish [7], [23], [33]. However, previous studies performed in fish showed contradictory results. On one hand, several studies showed that the proactive coping style is behaviourally characterised by a high level of locomotor activity during confinement or restraining as opposed to reactive individuals [7], [25], [38]. On the other hand, higher locomotor activity during confinement or restraining has been observed more in reactive as opposed to proactive individuals [23], [39]. It is interesting to notice that these latter studies showing higher locomotor activity during confinement in reactive animals used fish selected lines. In addition, proactive individuals usually exhibit a lower hypothalamus–pituitary–interrenal (HPI) axis reactivity. In the present study, no correlation between escape behaviour and plasma cortisol was found. Several studies have documented the lack of correlation between plasma cortisol levels obtained after stress and behavioural responses [7], [40], [41]. Some authors have suggested that cortisol and behavioural responses to stressors are linked to two independent dimensions of stable trait characteristics [42]. These authors suggested that the quality of the response to a challenging condition (coping style) is independent from the quantity of that response (stress reactivity). According to the same authors, the physiological responses to stress such as the HPI axis reactivity (one of the most significant differences between proactive and reactive individuals) is more related to an emotional response to stress than to coping styles. Eventually a decoupling of these axis, coping styles and emotional, could bring new light to understand the pronounced individual variation in plasma cortisol response observed in seabream after stress.

The other test that revealed consistent behavioural responses was the risk-taking test. Certain individuals were consistently the first to take the risk to venture into an unknown environment where food was present. One may wonder what the main driving force leading fish to cross the opening into a new environment was: 1) the willingness to explore a new environment; or 2) the motivation to eat, since food was only available in the new area. Toms et al. [21] suggested that hunger levels may influence risk-taking instead of proactive traits. In our study fish were fed *ad libitum* prior to the transfer to the risk-taking tank which could have minimized the differences in hunger level between proactive and reactive. On the other hand, proactive and reactive individuals differ in their metabolism [25], [43], consequently we cannot exclude that proactive individuals were hungrier and probably take more risks like going into a potentially dangerous or unknown environment, to get food.

Considering the consistency across contexts, individuals escaping more during the restraining test also escaped faster from hypoxia conditions. These results are in contrast to the findings of Laursen et al. [34], who reported that reactive fish escaped faster to hypoxic conditions. This suggests that reactive fish exhibit higher levels of behavioural flexibility. However, another study using the same selected trout lines suggested that depending on the context, proactive individual may adopt a more flexible behaviour [22]. One possibility to explain the differences found in the present study using seabream as compared to Laursen et al. [34] is the existence of species-specific differences in sub-lethal effects of reduced levels of dissolved oxygen; around 3 mg.L^−1^ in trout [44] and 1 mg.L^−1^ in seabream [45]. Therefore the propensity to escape could be expected to be different between these species. In Laursen et al. [34] individuals exhibit escape behaviour when exposed to decreased oxygen levels varying from 90 to 30% saturation. In seabream, however, individuals start escaping hypoxia only when oxygen concentrations reach level close to 30% saturation (3 mg.L^−1^). These differences in responsiveness may suggest that in trout, reactive individuals known to be more sensitive to changes in environmental conditions [6] are the first to escape hypoxia. However in seabream, escape behaviour starts only when oxygen concentrations reach to sub-lethal levels. In such situation, proactive individuals known to exhibit active attempts to counteract stressors [5] could be the first to escape hypoxia. To which extent the onset of responses of proactive and reactive individuals is dependent of how strong the stress is (or is interpreted to be) close to life-threatening conditions needs to be further investigated.

In this study, individuals exhibiting typical proactive characteristics (higher risk taking, higher escaping behaviour) were individuals taking longer to recover their feed intake while in isolation. These results are in contrast with [23] and [17] who showed a quicker recovery of feed intake in proactive as compared to reactive fish. However, other studies [6], [46], showed opposite results, i.e. proactive individual take longer to recover feed intake. Such inconsistency of results may be due to species-specific behaviour and/or to previous experiences (e.g. social experiences, nutritional background) that fish were exposed prior to the start of the experiments. In our study, reactive individuals recover feed intake faster and this can be due to showing some kind of compensatory feed intake as a result of previous social environment. Alternatively, reactive individuals by being more flexible [6] could have adapted faster when placed in a new environment.

In the present study the novel object test did not result in consistent behavioural responses. In contrast, [31] screened bold and shy individuals using their latency to come within close proximity of a novel object. However, [27] found a lack of consistency in exploration–avoidance traits as measured by the novel object test in Mozambique tilapia (*Oreochromis mossambicus*). In highly social species, personality traits may vary with social context and when this happens, it is crucial to take in account the social setting when assessing personality traits. Another possible explanation, for the absence of significant results in the novel object test could be related to differences in the size of the experimental glass aquarium. The experimental glass aquarium used in our study was square compared with rectangular tanks used by [31] and consequently in our study individuals could have had more difficulty to express exploration-avoidance behaviour towards the novel object, once they had less space available between aquarium walls and the object.

The present study shows for the first time a link between individually- and grouped- based test in fish personality characterization. Nearly all studies developed to study fish personality were based on individually-based tests [7], [23], [31], [33]. A few examples are available using grouped-based tests [34], [35]. However, to the best of our knowledge no study has used both approaches to assess personality in fish. One of the main criticisms with individually based tests is that they do not reflect what is happening in a group. On one hand different personalities could exhibit a different degree of sensitization to isolation. On the other hand group testing may lead to individuals modulating their own behaviour based on otheŕs behaviours (e.g. facilitation [28]). An interesting extension of the previous study would be to repeat with the same individuals the same test both in individual and grouped-based contexts and compare the behavioural responses.

In summary, this study suggests that individual differences in behavioural responses towards challenges reflect the presence of personality in fish. Using a non-selected fish line we found consistency over time and across-context in behavioural responses to challenges using individual and grouped-based tests. This study highlights the possibility to predict behaviour in groups from individual personality traits. Therefore, these findings may contribute to understand the pronounced individual variation in stress responses observed in this species. Furthermore, this study highlights the possibility to develop mass-screening methods to assess personality in fish that are grouped-based and therefore less time consuming as compared to individual-based tests.

## References

[1] Budaev SV, Zworykin DD (2002) Individuality in fish behavior: Ecology and comparative psychology. J Ichthyol 42: 189–195.

[2] Sih A, Bell AM, Johnson JC, Ziemba RE (2004) Behavioral syndromes: an integrative overview. Q Rev Biol 19: 372–378. 10.1086/422893 15529965

[3] Réale D, Dingemanse NJ, Kazem AJN, Wright J (2010) Evolutionary and ecological approaches to the study of personality. Phil Trans R Soc B 365: 3937–3946. 21078646 10.1098/rstb.2010.0222 PMC2992753

[4] Koolhaas JM, Korte SM, De Boer SF, Van Der Vegt BJ, Van Reenen CG, et al (1999) Coping styles in animals: current in behavior and stress-physiology. Neurosci Biobehav Rev 23: 925–935. 10580307 10.1016/s0149-7634(99)00026-3

[5] Benus RF, Bohus B, Koolhaas JM, van Oortmerssen GA (1991) Heritable variation for aggression as a reflection of individual coping strategies. Cell Mol Life Sci 47: 1008–1019. 10.1007/BF01923336 1936199

[6] Ruiz-Gomez ML, Huntingford FA, Øverli Ø, Thörnqvist P-O, Höglund E (2011) Response to environmental change in rainbow trout selected for divergent stress coping styles. Physiol Behav 102: 317–322. 21130105 10.1016/j.physbeh.2010.11.023

[7] Silva PIM, Martins CIM, Engrola S, Marino G, Øverli Ø, et al (2010) Individual differences in cortisol levels and behaviour of Senegalese sole (*Solea senegalensis*) juveniles: Evidence for coping styles. Appl Anim Behav Sci 124: 75–81.

[8] Øverli Ø, Sørensen C, Pulman KGT, Pottinger TG, Korzan W, et al (2007) Evolutionary background for stress- coping styles: Relationships between physiological, behavioral, and cognitive traits in non-mammalian vertebrates. Neurosci Biobehav Rev 31: 396–412. 17182101 10.1016/j.neubiorev.2006.10.006

[9] Sørensen C, Johansen IB, Øverli Ø (2013) Neural plasticity and stress coping in teleost fishes. Gen Comp Endocrinol 181: 25–34. 23274407 10.1016/j.ygcen.2012.12.003

[10] Johansen IB, Sørensen C, Sandvik GK, Nilsson GE, Höglund E, et al (2012) Neural plasticity is affected by stress and heritable variation in stress coping style. Comp Biochem Physiol D 7: 161–171. 10.1016/j.cbd.2012.01.002 22285148

[11] Huntingford F, Adams C (2005) Behavioural syndromes in farmed fish: implications for production and welfare. Behaviour 142: 1213–1227.

[12] Martins CIM, Galhardo L, Noble C, Damsgård B, Spedicato MT, et al (2011) Behavioural indicators of welfare in farmed fish. Fish Physiol Biochem38: 17–41. 10.1007/s10695-011-9518-8 PMC3276765 21796377

[13] Martins CIM, Conceição LEC, Schrama JW (2011) Feeding behavior and stress response explain individual differences in feed efficiency in juveniles of Nile tilapia *Oreochromis niloticus* . Aquaculture 312: 192–197.

[14] Martins CIM, Schaedelin FC, Mann M, Blum C, Mandl I, et al (2012) Exploring novelty: a component trait of behavioural syndromes in a colonial fish. Behaviour 149: 215–231. 10.1163/156853912X634430 PMC6485498 31031407

[15] Fevolden SE, Refstie T, Røed KH (1992) Disease resistance in rainbow trout (*Oncorhynchus mykiss*) selected for stress response. Aquaculture 104: 19–29.

[16] Fevolden SE, Nordmo R, Refstie T, Røed KH (1993) Disease resistance in Atlantic salmon (*Salmo salar*) selected for high or low responses to stress. Aquaculture 109: 215–224.

[17] Martins CIM, Conceição LEC, Schrama JW (2011) Consistency of individual variation in feeding behaviour and its relationship with performance traits in Nile tilapia *Oreochromis niloticus* . Appl Anim Behav Sci 133: 109–116.

[18] MacKenzie S, Ribas L, Pilarczyk M, Capdevila DM, Kadri S, et al (2009) Screening for coping style increases the power of gene expression studies. PLOS One 4: e5314. 19390591 10.1371/journal.pone.0005314 PMC2669184

[19] Alves RN, Cordeiro O, Silva TS, Richard N, Vareilles M, et al (2010) Metabolic molecular indicators of chronic stress in gilthead seabream (*Sparus aurata*) using comparative proteomics. Aquaculture 299: 57–66.

[20] Réale D, Reader SM, Sol D, McDougall PT, Dingemanse NJ (2007) Integrating animal temperament within ecology and evolution. Biol Rev 82: 291–318. 17437562 10.1111/j.1469-185X.2007.00010.x

[21] Toms CN, Echevarria DJ, Jouandot DJ (2010) A methodological review of personality-related studies in fish: Focus on the Shy-Bold axis of behavior. Int J Comp Psychol 23: 1–25.

[22] Basic D, Winberg S, Schjolden J, Krogdahl Å, Höglund E (2012) Context-dependent responses to novelty in Rainbow trout (*Oncorhynchus mykiss*), selected for high and low post-stress cortisol responsiveness. Physiol Behav 105: 1175–1181. 22226991 10.1016/j.physbeh.2011.12.021

[23] Øverli Ø, Sørensen C, Nilsson GE (2006) Behavioral indicators of stress-coping style in rainbow trout: Do males and females react differently to novelty? Physiol Behav 87: 506–512. 16455115 10.1016/j.physbeh.2005.11.012

[24] Barreto RE, Volpato GL (2011) Ventilation rates indicate stress-coping styles in Nile tilapia. J Bioscience 36: 851–855. 10.1007/s12038-011-9111-4 22116283

[25] Martins CIM, Castanheira MF, Engrola S, Costas B, Conceição LEC (2011) Individual differences in metabolism predict coping styles in fish. Appl Anim Behav Sci 130: 135–143.

[26] Galhardo L, Oliveira RF (2009) Psychological stress and welfare in fish. Annu Rev Biomed Sci 11: 1–20.

[27] Galhardo L, Vitorino A, Oliveira RF (2012) Social familiarity modulates personality trait in a cichlid fish. Biol Lett 8: 936–938. 22859562 10.1098/rsbl.2012.0500 PMC3497109

[28] Reebs SG (2000) Can a minority of informed leaders determine the foraging movements of a fish shoal? Anim Behav 59: 403–409. 10675263 10.1006/anbe.1999.1314

[29] Champagne DL, Hoefnagels CCM, de Kloet RE, Richardson MK (2010) Translating rodent behavioral repertoire to zebrafish (*Danio rerio*): Relevance for stress research. Behav Brain Res 214: 332–342. 20540966 10.1016/j.bbr.2010.06.001

[30] Barazi-Yeroulanos L (2010) Synthesis of Mediterranean marine finfish aquaculture – a marketing and promotion strategy. General Fisheries Commission for the Mediterranean. No. 88Rome: FAO 198p.

[31] Frost AJ, Winrow-Giffen A, Ashley PJ, Sneddon LU (2007) Plasticity in animal personality traits: does prior experience alter the degree of boldness? Proc R Soc B 274: 333–339. 10.1098/rspb.2006.3751 PMC1702388 17164196

[32] Arends RJ, Mancera JM, Muñoz JL, Bonga SEW, Flik G (1999) The stress response of the gilthead sea bream (*Sparus aurata* L.) to air exposure and confinement. J Endocrinol 163: 149–157. 10495417 10.1677/joe.0.1630149

[33] Martins CIM, Silva PIM, Conceição LEC, Costas B, Höglund E, et al (2011) Linking fearfulness and coping styles in fish. PLOS One 6: e28084. 22140511 10.1371/journal.pone.0028084 PMC3227632

[34] Laursen DC, Olsén HL, Ruiz-Gomez ML, Winberg S, Höglund E (2011) Behavioural responses to hypoxia provide a non-invasive method for distinguishing between stress coping styles in fish. Appl Anim Behav Sci 132: 211–216.

[35] Huntingford FA, Andrew G, Mackenzie S, Morera D, Coyle SM, et al (2010) Coping strategies in a strongly schooling fish, the common carp *Cyprinus carpio* . J Fish Biol 76: 1576–1591. 20557617 10.1111/j.1095-8649.2010.02582.x

[36] Irwin S, Kenny AP, O′Halloran J, FitzGerald RD, Duggan PF (1999) Adaptation and validation of a radioimmunoassay kit for measuring plasma cortisol in turbot. Comp Biochem Physiol C 124: 27–31. 10579645 10.1016/s0742-8413(99)00043-2

[37] Bolhuis JE, Schouten WGP, Schrama JW, Wiegant VM (2005) Individual coping characteristics, aggressiveness and fighting strategies in pigs. Anim Behav 69: 1085–1091.

[38] Brelin D, Petersson E, Winberg S (2005) Divergent stress coping styles in juvenile brown trout (*Salmo trutta*). Ann. N.Y. Acad. Sci 1040: 239–245. 10.1196/annals.1327.033 15891033

[39] Øverli Ø, Pottinger TG, Carrick TR, Øverli E, Winberg S (2002) Differences in behaviour between rainbow trout selected for high- and low-stress responsiveness. J Exp Biol 205: 391–395. 11854375 10.1242/jeb.205.3.391

[40] van Erp-van der Kooij E, Kuijpers AH, van Eerdenburg FJCM, Dieleman SJ, Blankenstein DM, et al (2003) Individual behavioural characteristics in pigs—influences of group composition but no differences in cortisol responses. Physiol Behav 78: 479–488. 12676285 10.1016/s0031-9384(03)00002-7

[41] van de Nieuwegiessen PG, Boerlage AS, Verreth JAJ, Schrama JW (2008) Assessing the effects of a chronic stressor, stocking density, on welfare indicators of juvenile African catfish, *Clarias gariepinus* Burchell. Appl Anim Behav Sci 115: 233–243.

[42] Koolhaas JM, de Boer SF, Coppens CM, Buwalda B (2010) Neuroendocrinology of coping styles: Towards understanding the biology of individual variation. Front Neuroendocrin 31: 307–321. 10.1016/j.yfrne.2010.04.001 20382177

[43] Careau V, Thomas D, Humphries MM, Réale D (2008) Energy metabolism and animal personality. Oikos 117: 641–653.

[44] Raleigh RF, Hickman T, Solomon RC, Nelson PC (1984) Habitat suitability information: rainbow trout U.S. Fish Wildl. Serv. FWS/OBS-82/10.60. 64 p.

[45] Reig AC (2001) Influencia de la temperatura y la salinidad sobre el crescimiento y consumo de oxígeno de la Dorada (*Sparua aurata* L.). Barcelona:Universitat de Barcelona 206 p.

[46] LeBlanc S, Höglund E, Gilmour KM, Currie S (2012) Hormonal modulation of the heat shock response: insights from fish with divergent cortisol stress responses. Am J Physiol 302: R184–R192. 10.1152/ajpregu.00196.2011 22031780

